# Association of novel *ERLIN2* gene variants with hereditary spastic paraplegia

**DOI:** 10.1038/s41439-024-00305-9

**Published:** 2025-01-06

**Authors:** R. Bermejo Ramírez, N. Villena Gascó, L. Ruiz Palmero, G. A. Ribes Bueno, E. S. Yamanaka, J. Piqueras Flores, J. M. Flores Barragán, E. Buces González, J. D. Arroyo Andújar

**Affiliations:** 1 Progenie Molecular S.L.U, Valencia, Spain; 2 Inherited Cardiomyopathies Unit, Cardiology Department, Ciudad Real University General Hospital, Ciudad Real, Spain; 3 Faculty of Medicine of Ciudad Real, University of Castilla-La Mancha, Ciudad Real, Spain; 4 Castilla-La Mancha Institute for Health Research (IDISCAM), Ciudad Real, Spain; 5 Neurology Department, Ciudad Real University General Hospital, Ciudad Real, Spain; 6 Clinical Analysis Department, Ciudad Real University General Hospital, Ciudad Real, Spain

**Keywords:** Diagnostic markers, Genetic association study, Neuromuscular disease

## Abstract

Two *ERLIN2* variants (NM_007175.8:c.660delA and NM_007175.8:c.869C>T) were detected in a Spanish patient with hereditary spastic paraplegia via whole-exome sequencing and software-based pathogenic variant selection. Segregation analysis revealed that the patient’s two affected siblings carried both variants, whereas their offspring, carrying only one variant, were asymptomatic, indicating the autosomal recessive nature of the disease. These findings suggest that the identified variants can be classified as pathogenic when they are present as compound heterozygous variants.

Hereditary spastic paraplegia (HSP) is one of the most heterogeneous neurological disorders, with weakness and spasticity of the lower extremities as the predominant manifestations^1^. However, HSP patients can exhibit high variability in the degree of severity, age of symptom onset and progression^2^. HSP also has a vast genetic background, with 84 loci and 67 causative genes identified for HSP to date^3,4^.

Variants in the endoplasmic reticulum lipid raft-associated protein 2 (*ERLIN2*) gene have been identified as the cause of spastic paraplegia type 18 (SPG18, OMIM 611225), which can be inherited in both autosomal recessive and autosomal dominant forms^5^. The autosomal recessive form is generally characterized by onset of progressive spastic paraplegia in early childhood, resulting in motor disability. Affected individuals show progressive tightening of the lower and upper extremities but can also exhibit seizures, multiple joint contractures, speech problems, intellectual disability and motor dysfunction^1,6^. On the other hand, the dominant inheritance usually leads to a pure form of HSP, with manifestations limited to neuromotor symptoms^2^.

In this report, we describe two *ERLIN2* variants identified in a Spanish family affected by autosomal recessive HSP. The index case was a 61-year-old Spanish male diagnosed with HSP who started showing neuromuscular symptoms at age 25. He presented with clubfoot, lower limb spasticity, hypertonia and a progressively affected gait. Currently, the patient is functionally dependent for performing daily tasks, requires a wheelchair for locomotion and suffers from impaired sphincter control. He has also been diagnosed with non-ischemic dilated cardiomyopathy with moderate left ventricular systolic dysfunction. Sustained monomorphic ventricular tachycardia at age 32 required the implantation of a cardioverter-defibrillator. Owing to this device, performing magnetic resonance imaging (MRI) scans was not possible. No genetic markers related to dilated cardiomyopathy were detected in a 112-gene panel. The patient has two siblings, one male and one female, who were also diagnosed with HSP and presented with similar lower-limb neuromotor symptoms. Their parents were not studied but did not exhibit HSP symptoms.

The proband’s brother was 64 years old, presented symptom onset at age 28 and could walk with the aid of crutches. Lumbar MRI revealed L3 to S1 discopathy, with right posterior disc extrusion at L5-S1, affecting the S1 nerve root at the ipsilateral foraminal recess. Additionally, this patient was diagnosed with ischemic angina-free cardiopathy with one-vessel disease and a moderately depressed left ventricular ejection fraction. A drug-eluting stent was implanted in the circumflex artery due to an ST-elevation myocardial infarction event at age 56.

The proband’s 60-year-old sister presented the first symptoms at age 22 and currently requires a wheelchair for ambulation. She was also diagnosed with mild bilateral carpal tunnel syndrome and chronic tension-type headache, with no cardiac pathologies. No abnormalities were detected via brain MRI, apart from diffuse hyperintensities of a nonspecific nature in the white matter and a choroidal fissure cyst exhibiting signal characteristics similar to those of the cerebrospinal fluid. Cervical (C5-C6) and lumbar (L5-S1) disc herniations were found via lumbar MRI.

Candidate pathogenic variants were detected via whole-exome sequencing and selected after software filtering with the BioVisor© NGS (Progenie Molecular, Valencia, Spain). Sanger sequencing was performed to confirm the mutations and detect the variants in the family members used in the subsequent segregation analysis. A total of eight family members were included in the study: the 3 siblings and their unaffected offspring. The detailed procedure and electropherograms are presented in the Supplementary Information.

Through exome sequencing and the subsequent variant selection, two variants present in compound heterozygosity were identified in the *ERLIN2* gene (Fig. 1); these variants were suspected to cause the clinical findings in this family. The first variant is a frameshift variant located in exon 10, NM_007175.8:c.660delA, and the second variant is a missense variant located in exon 12, NM_007175.8:c.869C>T (rs759417913). To date, neither of these variants have been associated with HSP.

**Fig. 1:**
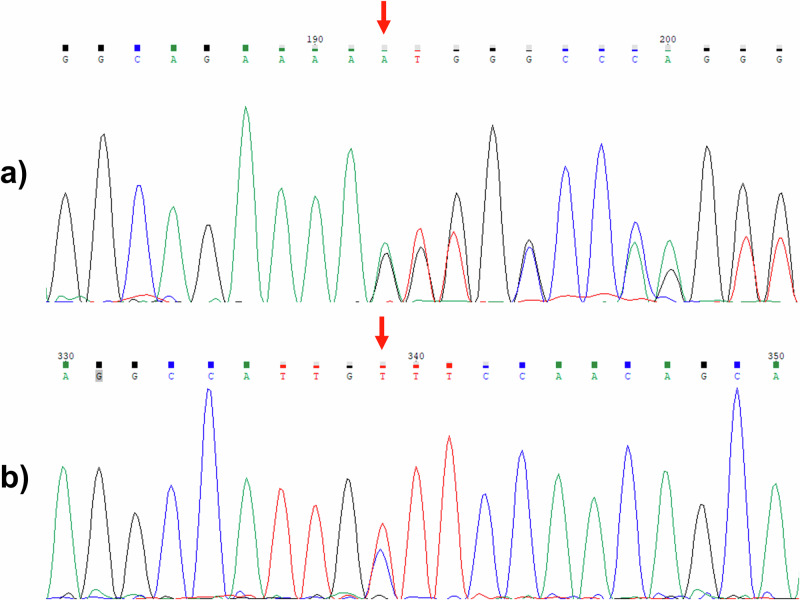
Sanger sequencing electropherograms of the identified *ERLIN2* variants (NM_007175.8) in a patient with hereditary spastic paraplegia. **a** c.660delA and **b** c.869C>T. Owing to the frameshift, the nucleotide call after this variant represents the overlapping sequences of the wild-type and mutant alleles. The red arrow indicates the variant.

c.660delA is an unreported deletion that causes a frameshift leading to a premature stop codon (NM_007175.8(NP_009106.1):p.(Val221Trpfs*13)). This variant had a very low reported frequency of 6.846 ×10^−7^ in gnomAD and was absent in the 1000 Genomes (1 K) and Human Genome Diversity Project (HGDP) databases. Although functional studies have not been carried out, the frameshift caused by this variant may lead to nonsense-mediated decay of the mRNA, resulting in a null effect. The SIFT indel tool^7^ predicted that this variant is damaging, with a confidence score of 0.529.

The missense variant c.869C>T is predicted to cause an alanine to valine substitution at position 290 (NM_007175.8(NP_009106.1):p.(Ala290Val)). The reported frequency for this variant was 2.045 ×10^−5^ in gnomAD, and it was not found in the 1 K or HGDP databases. This variant was predicted to be probably damaging by PolyPhen-2^8^ (score, 0.938) and deleterious by SIFT^9^ (score, 0).

Variants in the *ERLIN2* gene are associated with HSP type 18 (SPG18) and were first identified in patients with complicated autosomal recessive forms of the disease and early symptom onset^1,6,10^. However, a number of groups have also identified *ERLIN2* variants in patients with pure autosomal dominant forms with variable onset^3,5,11–15^. Other recent studies detected compound heterozygous *ERLIN2* variants in pure HSP patients: c.108A>T + c.395C>T in a 17-year-old patient with normal MRI findings and spasticity onset at age 3^16^ and c.481C>A + c.866T>C in a 67-year-old patient with onset at age 10 and normal MRI results^17^. In the present work, the three affected siblings presented predominant HSP manifestations; individually, the siblings presented other symptoms, such as sphincter control impairment, discopathies, carpal tunnel syndrome and chronic tension-type headache, indicating a complicated form of HSP. However, symptom onset was significantly later than that of other autosomal recessive HSPs, and no cognitive impairment was observed, suggesting that the direct association of phenotype with inheritance mechanisms is not yet clear in SPG18.

Two of the affected subjects in this study also manifested cardiological conditions. The index patient has idiopathic cardiomyopathy, which was not previously reported in SPG18 cases, although it was identified in SPG79^18^ and mitochondrial-DNA-related HSPs^19,20^. However, it is not clear if this condition is related to HSP or other causes; further investigation is necessary to evaluate the relationship.

Sanger sequencing of the candidate variants revealed that the three affected siblings carried both *ERLIN2* variants in compound heterozygosity, whereas their unaffected children carried only one variant and, to date, have not exhibited any phenotype linked to HSP pathology (Fig. 2). Age plays an important role in HSP, with affected individuals experiencing symptom onset at a relatively late age. For this reason, there is a minor possibility that the symptoms could manifest in third-generation individuals, especially those carrying the c.660delA variant, which is predicted to be the most damaging variant. However, subjects III-1 and III-2 have already reached the onset age of their parents and uncles but have not shown any symptoms, and their grandparents are asymptomatic. Thus, there is a strong probability that SPG18 in this family has autosomal recessive inheritance and that no HSP symptoms should be expected in monoallelic carriers.

**Fig. 2:**
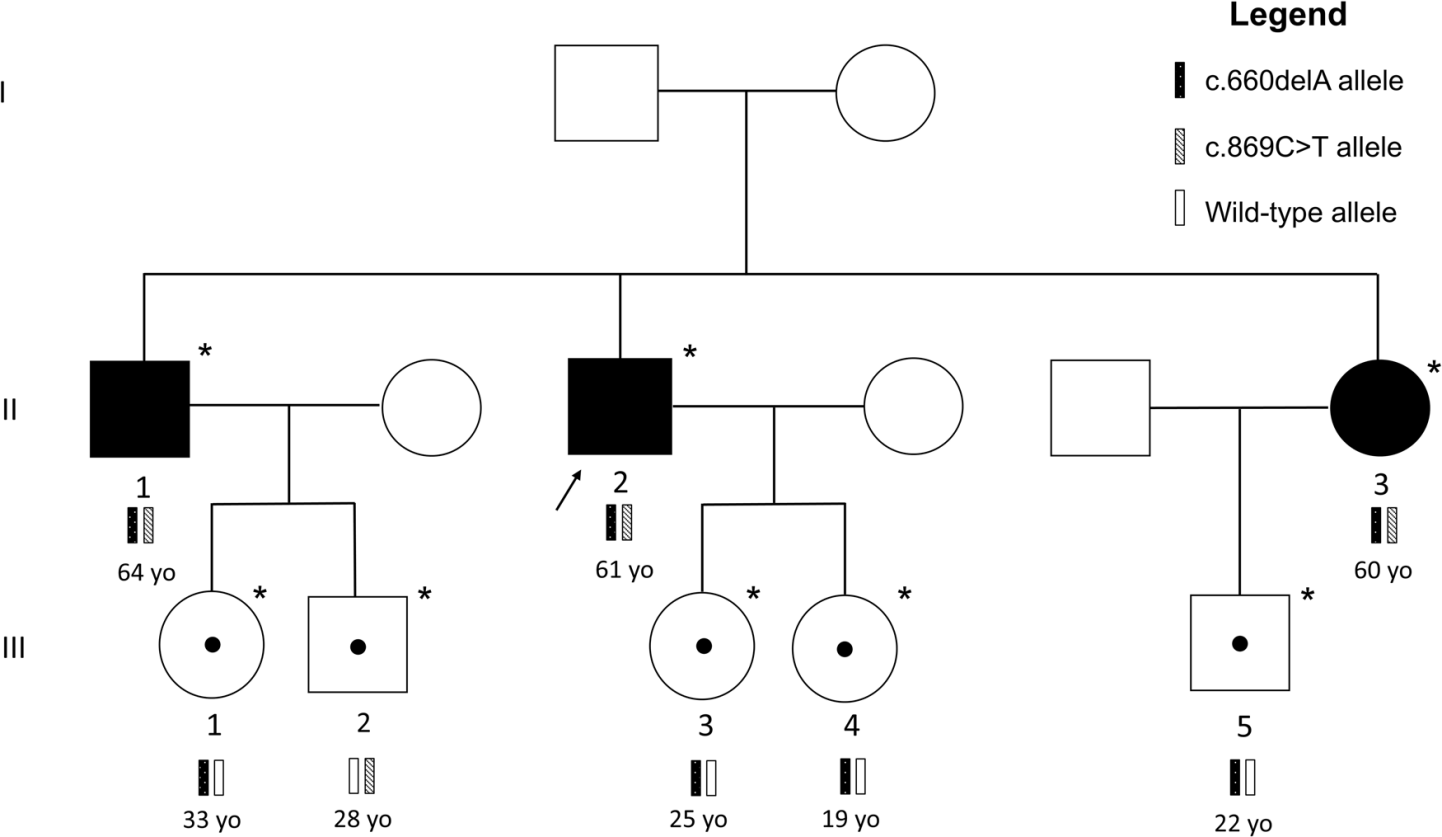
Family pedigree of a hereditary spastic paraplegia type 18 patient with *ERLIN2* variants (NM_007175.8). Subject II-2 is the index case. Subjects II-1 and II-3 are affected and carry both variants (c.660delA and c.869C>T). The tested offspring (daughters, nephews and niece of the index case) are all unaffected and carry only one variant. *Genotype validated by Sanger sequencing; square: male; circle: female; white-marked: unaffected; black-marked: affected; dot-marked: unaffected carrier.

In summary, the in silico and segregation analyses reinforce the pathogenic significance of both *ERLIN2* variants when present in compound heterozygosity and corroborate the autosomal recessive inheritance of HSP in this family. On the basis of these findings, we conclude that these variants can be considered pathogenic for SPG18, broadening the genetic spectrum of type 18 HSP and facilitating the diagnosis of similar cases in the future. Considering the presented results, we encourage the inclusion of the described *ERLIN2* variants in the routine diagnosis of HSP and other related neuromuscular diseases.

## HGV database

The relevant data from this Data Report are hosted at the Human Genome Variation Database at: 10.6084/m9.figshare.hgv.3474. 10.6084/m9.figshare.hgv.3477.

## Supplementary information

Supplementary Information

## Data Availability

The data that support the findings of this study are available from the corresponding author upon reasonable request. The NM_007175.8:c.660delA and NM_007175.8:c.869C>T variants were submitted to the ClinVar database under accession numbers VCV002663863.1 and VCV002663843.1.

## References

[1] Alazami, A. M., Adly, N., Al Dhalaan, H. & Alkuraya, F. S. A nullimorphic ERLIN2 mutation defines a complicated hereditary spastic paraplegia locus (SPG18). *Neurogenetics* **12**, 333–336 (2011). 21796390 10.1007/s10048-011-0291-8 PMC3215864

[2] Fink, J. K. Hereditary spastic paraplegia: clinico-pathologic features and emerging molecular mechanisms. *Acta Neuropathol.* **126**, 307–328 (2013). 23897027 10.1007/s00401-013-1115-8 PMC4045499

[3] Rydning, S. L. et al. A novel heterozygous variant in ERLIN2 causes autosomal dominant pure hereditary spastic paraplegia. *Eur. J. Neurol.* **25**, 943–e71 (2018). 29528531 10.1111/ene.13625

[4] Tesson, C., Koht, J. & Stevanin, G. Delving into the complexity of hereditary spastic paraplegias: how unexpected phenotypes and inheritance modes are revolutionizing their nosology. *Hum. Genet.* **134**, 511–538 (2015). 25758904 10.1007/s00439-015-1536-7 PMC4424374

[5] Park, J. M. et al. An autosomal dominant ERLIN2 mutation leads to a pure HSP phenotype distinct from the autosomal recessive ERLIN2 mutations (SPG18). *Sci. Rep.* **10**, 1–6 (2020). 32094424 10.1038/s41598-020-60374-y PMC7039913

[6] Yildirim, Y. et al. A frameshift mutation of ERLIN2 in recessive intellectual disability, motor dysfunction and multiple joint contractures. *Hum. Mol. Gen.* **20**, 1886–1892 (2011). 21330303 10.1093/hmg/ddr070

[7] Hu, J. & Ng, P. C. SIFT Indel: predictions for the functional effects of amino acid insertions/deletions in proteins. *PloS One* **8**, e77940 (2013). 24194902 10.1371/journal.pone.0077940 PMC3806772

[8] Adzhubei, I., Jordan, D. M. & Sunyaev, S. R. Predicting functional effect of human missense mutations using PolyPhen‐2. *Curr. Protoc. Hum. Genet* **76**, 7–20 (2013). 10.1002/0471142905.hg0720s76 PMC4480630 23315928

[9] Ng, P. C. Henikoff S. SIFT: Predicting amino acid changes that affect protein function. *Nucleic Acids Res.* **31**, 3812–3814 (2003). 12824425 10.1093/nar/gkg509 PMC168916

[10] Wakil, S. M. et al. A novel splice site mutation in ERLIN2 causes hereditary spastic paraplegia in a Saudi family. *Eur. J. Med. Genet.* **56**, 43–45 (2013). 23085305 10.1016/j.ejmg.2012.10.003

[11] Tian, W. T. et al. Novel mutations in endoplasmic reticulum lipid raft-associated protein 2 gene cause pure hereditary spastic paraplegia type 18. *Chin. Med. J.* **129**, 2759–2761 (2016). 27824013 10.4103/0366-6999.193444 PMC5126172

[12] Srivastava, S. et al. Expansion of the genetic landscape of ERLIN2‐related disorders. *Ann. Clin. Transl. Neurol.* **7**, 573–578 (2020). 32147972 10.1002/acn3.51007 PMC7187699

[13] Chen, S. et al. More autosomal dominant SPG18 cases than recessive? The first AD‐SPG18 pedigree in Chinese and literature review. *Brain Behav.* **11**, e32395 (2021). 34734492 10.1002/brb3.2395 PMC8671789

[14] Trinchillo, A. et al. Expanding SPG18 clinical spectrum: autosomal dominant mutation causes complicated hereditary spastic paraplegia in a large family. *Neurol. Sci.* **45**, 4373–4381 (2024). 38607533 10.1007/s10072-024-07500-0 PMC11306645

[15] Wang, J. et al. A novel autosomal dominant ERLIN2 variant activates endoplasmic reticulum stress in a Chinese HSP family. *Ann. Clin. Transl. Neur* **10**, 2139–2148 (2023). 10.1002/acn3.51902 PMC10646992 37752894

[16] Travaglini, L. et al. The impact of next-generation sequencing on the diagnosis of pediatric-onset hereditary spastic paraplegias: new genotype-phenotype correlations for rare HSP-related genes. *Neurogenetics* **19**, 111–121 (2018). 29691679 10.1007/s10048-018-0545-9

[17] Cioffi, E. et al. Hereditary spastic paraparesis type 18 (SPG18): new ERLIN2 variants in a series of Italian patients, shedding light upon genetic and phenotypic variability. *Neurol. Sci.* **45**, 3845–3852 (2024). 38427163 10.1007/s10072-024-07423-w PMC11255072

[18] McMacken, G. et al. Behr syndrome and hypertrophic cardiomyopathy in a family with a novel UCHL1 deletion. *J. Neurol.* **267**, 3643–3649 (2020). 32656641 10.1007/s00415-020-10059-3 PMC7674332

[19] Corona, P. et al. Novel heteroplasmic mtDNA mutation in a family with heterogeneous clinical presentations. *Ann. Neurol.* **51**, 118–122 (2002). 11782991 10.1002/ana.10059

[20] Verny, C. et al. Hereditary spastic paraplegia-like disorder due to a mitochondrial ATP6 gene point mutation. *Mitochondrion* **11**, 70–75 (2011). 20656066 10.1016/j.mito.2010.07.006

