# Supplementary material for: Association of novel ERLIN2 gene variants with hereditary spastic paraplegia
Source: Hum Genome Var. 2025 Jan 6;12:3. doi: 10.1038/s41439-024-00305-9 (PMC11704067; doi:10.1038/s41439-024-00305-9)
Supplement: Supplementary file 1 — Supplementary Information [file 41439_2024_305_MOESM1_ESM.pdf]

Association of novel *ERLIN2* gene variants to hereditary spastic paraplegia

R. Bermejo Ramírez,[1] N. Villena Gascó,[1] L. Ruiz Palmero,[1] G.A. Ribes Bueno,[1] E.S. Yamanaka,[1] J. Piqueras Flores,[2,3,4] J.M. Flores Barragán,[5] E. Buces González,[6] J. D. Arroyo Andújar[1,]*

[1] Progenie Molecular S.L.U. Valencia, Spain.

[2] Unidad de Cardiopatías Familiares, Hospital General de Ciudad Real. Ciudad Real, Spain.

[3] Facultad de Medicina de Ciudad Real. Universidad de Castilla La Mancha. Ciudad Real, Spain.

[4] Instituto de Investigación Biomédica del Sescam (IDISCAM). Ciudad Real, Spain.

[5] Servicio de Neurología. Hospital General de Ciudad Real. Ciudad Real, Spain.

[6] Servicio de Análisis Clínicos. Hospital General de Ciudad Real. Ciudad Real, Spain.

*Corresponding author. Progenie Molecular S. L.U. Edificio Progenie. Calle Valle de la Ballestera 56, 46015 Valencia, Spain. E-mail address: darroyo@progenie-molecular.com

SUPPLEMENTARY INFORMATION

DNA was purified from a patient's peripheral blood sample collected in EDTA tubes. DNA purification was carried out on a MagCore® Super extractor instrument (RBC Bioscience Corp., New Taipei City, Taiwan) using a MagCore® Viral Nucleic Acid extraction kit (Low PCR inhibition MVN400-04).

Whole-exome sequencing was performed using the SureSelect Human All Exon V6 (Agilent Technologies, Santa Clara, California, USA) capture kit and Illumina HiSeq 4000 platform. The mean depth (dp) of target regions of the exome was 81.8X. A next-generation sequencing (NGS) targeted panel (Chart S1) was used to analyse 65 genes related to hereditary spastic paraplegia (HSP) in the proband sample with 96.31% of the target region with a dp≥20X.

Chart S1. HSP-associated genes included in the NGS targeted panel.

ALDH18A1	*C12orf65*	*FA2H*	*MAG*	*SPG11*
AMPD2	*C19orf12*	*FARS2*	*NIPA1*	*SPG20*
AP4B1	*CAPN1*	*GAD1*	*NT5C2*	*SPG21*
AP4E1	*CCT5*	*GBA2*	*PGAP1*	*SPG7*
AP4M1	*CPT1C*	*HACE1*	*PLP1*	*TECPR2*
AP4S1	*CYP2U1*	*IBA57*	*PNPLA6*	*TFG*
AP5Z1	*CYP7B1*	*KDM5C*	*REEP1*	*UCHL1*
ARL6IP1	*DDHD1*	*KIDINS220*	*REEP2*	*USP8*
ARSI	*DDHD2*	*KIF1A*	*RTN2*	*VPS37A*
ATL1	*DSTYK*	*KIF1C*	*SLC16A2*	*WASHC5*
ATP13A2	*ENTPD1*	*KIF5A*	*SLC33A1*	*WDR48*
B4GALNT1	*ERLIN1*	*KLC2*	*SNTB1*	*ZFYVE26*
BSCL2	*ERLIN2*	*L1CAM*	*SPAST*	*ZFYVE27*

Variant filtering, analysis and interpretation of the data was performed using the BioVisor© NGS software (Progenie Molecular, Valencia, Spain) and its own pipeline method, applying the following criteria in order to discard non-pathogenic variants: variants with frequency ≥ 0,01; benign variants according to ClinVar data; intronic variants located within ±2 nt; 5´UTR variants located within -2 nt; 3´UTR variants located within + 2 nt; variants in non-coding exons; synonymous variants.

The single nucleotide variants of interest were validated by Sanger sequencing with a BigDye Terminator® kit (Applied Biosystems, USA), an ABI PRISM® 310 Genetic Analyzer (Applied Biosystems) and a SeqStudio Genetic Analyzer (Applied Biosystems). Sanger sequencing was performed for the proband and seven relatives to carry out the segregation analysis. Figures S1 and S2 show the Sanger electropherograms for the 7 relatives tested for NM_007175.8:c.660delA and NM_007175.8:c.869C>T variants in the *ERLIN2* gene, respectively.

The information about the genetic variants was reviewed from the published literature, variant databases, as well as online genetics resources (OMIM®, GeneReviews®, ClinVar, LitVar).

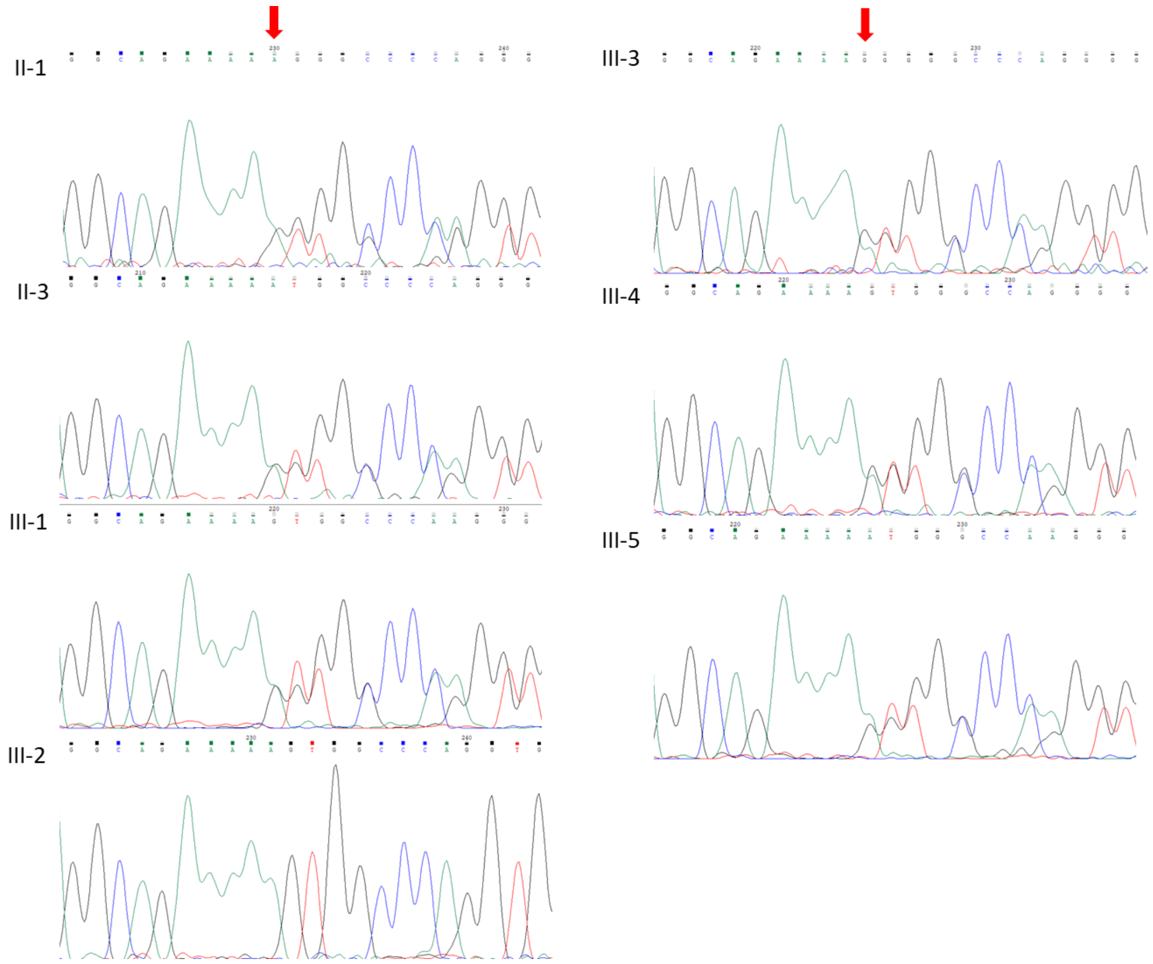

Figure S1 Sanger sequencing electropherograms of the *ERLIN2* variant NM_007175.8:c.660delA in the family members of a hereditary spastic paraplegia patient. Excepting for subject III-2, all members present this variant in heterozygosity. Due to the frameshift, the nucleotide-call after this variant represents the overlapped sequences wild-type and mutant alleles. A red arrow indicates the variant.

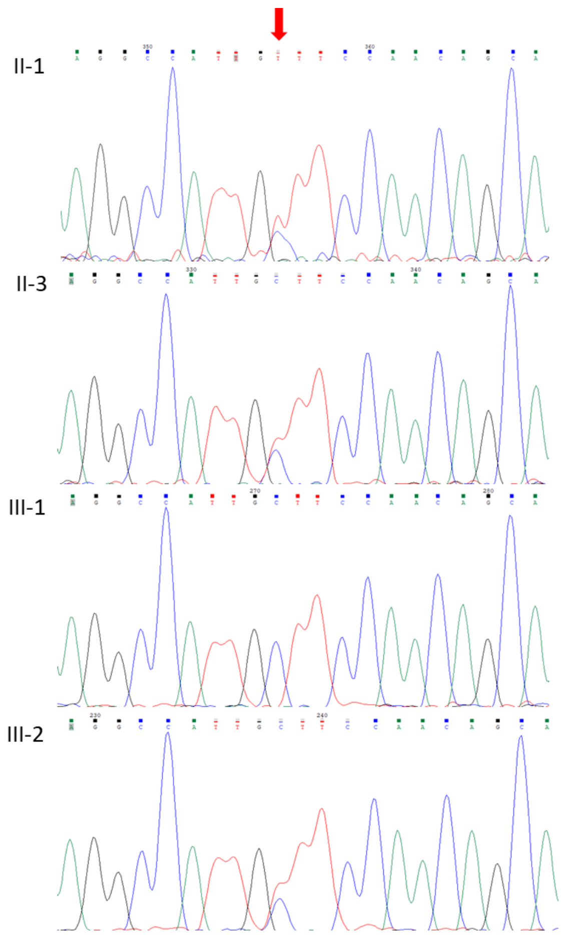

Figure S2 Sanger sequencing electropherograms of the *ERLIN2* variant NM_007175.8:c.869C>T in the family members of a hereditary spastic paraplegia patient. The variant was detected in subjects II-1, II-3 and III-2 in heterozygosity. A red arrow indicates the variant.
